# MPNFS-based integrative psychological intervention for adolescents with anxiety disorders: effects on anxiety, self-concept, parenting style, family intimacy, and social support

**DOI:** 10.3389/fpsyt.2026.1748651

**Published:** 2026-04-01

**Authors:** Liming Tan, Weihua Zhou

**Affiliations:** 1 Department of Nursing, Medical College of Jishou University, Jishou, Hunan, China; 2 Prenatal Diagnosis Center of Xiangxi Tujia and Miao Autonomous Prefecture People’s Hospital (The First Affiliated Hospital of Jishou University), Jishou, Hunan, China

**Keywords:** adolescent, anxiety disorder, family functioning, integrative psychological intervention, MPNFS model, quality of life

## Abstract

**Objective:**

This study aimed to evaluate the efficacy of an MPNFS-based integrative psychological intervention for adolescents with anxiety disorders, focusing on anxiety symptoms, Self-Concept, Parenting Style, Family Intimacy, and Social Support.

**Methods:**

A retrospective study was conducted on 196 adolescents (10–18 years) with anxiety disorders treated at Xiangxi Tujia and Miao Autonomous Prefecture People’s Hospital from January 2024 to October 2025. Among them, 89 received routine outpatient care and 107 received a 6-week MPNFS-based integrative program as part of routine clinical care. Clinical outcomes included the Self-Rating Anxiety Scale (SAS), Self-Rating Depression Scale (SDS), Piers-Harris Children’s Self-Concept Scale (PHCSS), Egna Minnen av Barndoms Uppfostran (EMBU), Family Adaptability and Cohesion Scale II Chinese Version (FACESII-CV), Perceived Social Support Scale (PSSS), and Short Form 36 Health Survey (SF-36).

**Results:**

Baseline characteristics were comparable between groups. After 6 weeks, the observation group demonstrated significantly greater improvements than the control group in anxiety (SAS: estimate = −7.5, 95% CI −8.6 to −6.4, *P* < 0.001) and depression (SDS: −7.4, 95% CI −8.5 to −6.3, *P* < 0.001). Self-concept improved across all PHCSS subscales (Behavior, Intellectual/School Status, Physical Appearance, Anxiety, Popularity, Happiness; total score: 18.0, 95% CI 16.9–19.1, *P* < 0.001). Parenting factors improved, including increased paternal emotional warmth (10.1, 95% CI 9.1–11.1, *P* < 0.001) and maternal emotional warmth (0.8, 95% CI 0.2–1.4, *P* = 0.012), along with reduced paternal punishment and rejection, and maternal over-interference/protection (all *P* < 0.001). Family functioning (FACESII-CV: 47.4, 95% CI 46.1–48.7, *P* < 0.001), perceived social support (PSSS: 20.2, 95% CI 19.0–21.4, *P* < 0.001), and all SF-36 domains showed significantly greater improvements in the observation group (all *P* < 0.001).

**Conclusion:**

The MPNFS-based integrative intervention effectively alleviates anxiety and depression, enhances self-concept, optimizes family functioning, strengthens social support, and improves quality of life in adolescents with anxiety disorders. These findings highlight the clinical potential of a structured multi-component approach and provide evidence supporting its adoption in routine outpatient care, while future longitudinal and randomized studies are needed to confirm the sustainability of these effects.

## Introduction

Adolescent anxiety disorder is one of the most prevalent psychiatric conditions during adolescence, characterized by excessive, persistent tension and uncontrollable worry that extends beyond typical, age-appropriate concerns (1). Epidemiological and clinical evidence confirms its high recurrence rate; without targeted intervention, the disorder often progresses into a chronic, remitting-relapsing course, significantly increasing the risk of comorbid mental health conditions, such as major depressive disorder, obsessive-compulsive disorder, and substance use disorders later in life.

In addition to core anxiety symptoms, adolescents with this disorder often exhibit interconnected psychosocial impairments: markedly reduced family intimacy—defined as emotional closeness, effective communication, and mutual support among family members—and insufficient perceived social support, which includes inadequate backing from family, peers, and community networks (2). These factors not only exacerbate acute anxiety but also hinder functional recovery, creating a self-perpetuating cycle that impairs academic performance, interpersonal relationships, and long-term psychological well-being (3). Despite growing recognition of these psychosocial factors, existing interventions frequently target either symptom relief or family/social functioning, rarely addressing both in a structured, multi-component framework. Thus, there is an urgent need for comprehensive, integrative interventions that simultaneously target symptomatic and psychosocial dimensions of adolescent anxiety.

Previous research has increasingly highlighted the effectiveness of integrative psychological interventions in adolescents, which combine cognitive-behavioral therapy, family therapy, and social support mobilization to address both psychological symptoms and psychosocial functioning (4, 5). Studies indicate that multi-component interventions often yield better outcomes than single-focus treatments, particularly in enhancing self-concept, family cohesion, and perceived social support (6, 7).

The MPNFS (Medication, Psychological intervention, Nursing, Family care, Social support) model represents a structured, evidence-based framework for delivering such comprehensive care (8). Unlike traditional approaches that primarily focus on pharmacotherapy or isolated psychological counseling, the MPNFS model systematically integrates five synergistic components: tailored pharmacotherapy for symptom management, structured psychological interventions for cognitive and emotional regulation, standardized nursing care to monitor adherence and well-being, family-centered approaches to optimize relational dynamics, and organized social support mobilization to enhance external resources (9). Previous studies have demonstrated the efficacy of the MPNFS model in adult and clinical populations, including post-surgical neurological recovery and stress reduction in healthcare workers, highlighting its flexibility and modular design for adapting interventions to individual needs (10, 11). However, its application in adolescents with anxiety disorders remains unexplored, representing a clear research gap.

Building on this evidence, the present retrospective study examines the MPNFS-based integrative intervention, implemented from January 2024 according to patient and family preference, clinical judgment, and available resources, in adolescents with anxiety disorders at our hospital. We aimed to evaluate whether this approach is associated with reductions in anxiety and depressive symptoms, as well as improvements in self-concept, family intimacy, and perceived social support, compared with routine outpatient care. By situating our work within the existing literature on multi-component interventions and explicitly describing the rationale for adopting the MPNFS model, this study provides real-world evidence on a structured, multi-level intervention strategy for adolescent mental health, advancing both theoretical understanding and clinical practice.

## Methods

### Study design and participants

This retrospective comparative cohort study reviewed 196 adolescents diagnosed with anxiety disorders who attended Xiangxi Tujia and Miao Autonomous Prefecture People’s Hospital from January 2024 to October 2025. All data were extracted from the hospital’s electronic medical record system and psychological assessment archives as part of usual clinical practice. Data were collected on patients who had received routine outpatient care (n=89) or the MPNFS-based integrative psychological program (n=107) as part of standard clinical practice. The choice of intervention was based on clinical decision-making, taking into account patient and family preference, clinician judgment, and resource availability; no randomization procedure was applied. To reduce baseline imbalance and confounding, strict inclusion and exclusion criteria were applied, and baseline demographic and clinical characteristics were comprehensively compared between groups.

The study was approved by the Ethics Committee of Jishou University(No.2023007). Given the retrospective design and use of anonymized data, the requirement for written informed consent was waived by the ethics committee.

Inclusion criteria: ①Diagnosed with anxiety disorders by specialist psychologists in accordance with the Chinese Classification of Mental Disorders, 3rd Edition (CCMD-3); ②Aged 10–18 years; ③Complete clinical data available.
Exclusion criteria: ①Comorbid organic mental disorders, mental disorders due to psychoactive or non-addictive substances, schizophrenia, depressive disorder, or bipolar affective disorder; ②Severe physical illnesses; ③Active suicidal ideation.

### Sample size and statistical power

Sample size estimation was conducted *a priori* based on the primary outcome, Zung’s Self-Rating Anxiety Scale (SAS) score. Standard deviation (SD) estimates were informed by normative data from previous research on SAS, which reported SDs of approximately 6–10 points in clinical and community samples (12). A minimal clinically important difference (MCID) of 5 points was assumed based on distribution-based methods (e.g., half a standard deviation), consistent with prior work on interpreting clinically meaningful changes in anxiety measures (13). Assuming a two-sided significance level (α) of 0.05 and a power (1 − β) of 0.80, the required sample size per group was calculated to detect the MCID with adequate statistical power. Given these parameters, a minimum of 41 participants per group was required.

### Intervention methods of control group

Participants received routine outpatient management for mental disorders. Specialist psychologists conducted systematic diagnosis and illness profiling, followed by psychoeducation on anxiety disorders, which covered etiological factors, symptom manifestations, treatment options, and prognosis. Pharmacotherapy was prescribed based on symptom severity (e.g., selective serotonin reuptake inhibitors for moderate-to-severe anxiety), with dosage adjustments made during follow-up visits, scheduled every 2 weeks. Participants were reminded of medication adherence and upcoming appointments through telephone reminders 3 days prior to each visit.

### Intervention methods of observation group

The observation group received MPNFS (Medication, Psychological intervention, Nursing, Family care, Social support)-based integrative psychological intervention. Each component of the MPNFS model was predefined, protocol-driven, and delivered in a standardized manner to ensure intervention fidelity and reproducibility.

#### Intervention team composition

The team was led by a psychiatric head nurse with a National Level-2 Psychological Counselor certificate and over 5 years of clinical experience in psychological intervention. The team also included 1 specialist psychologist, 4 psychiatric nurses with National Level-3 Psychological Counselor certificates, and 1 full-time psychologist. Role assignments were as follows: the specialist psychologist was responsible for pharmacotherapy and basic psychoeducation (consistent with the control group); 1 psychiatric nurse managed questionnaire distribution and data collection; 3 psychiatric nurses provided one-on-one individual psychological counseling; the full-time psychologist conducted family group psychotherapy and family psychological knowledge workshops; the head nurse supervised the evaluation of intervention efficacy, monitored adherence to the intervention protocol, and ensured fidelity of the program through regular team meetings and review of session records.

#### Intervention timeline and setting

The total intervention duration was 6 weeks.

Outpatient management by the specialist psychologist: conducted in the outpatient clinic, once weekly, 10–15 min per session.
Individual psychological counseling: delivered in the psychological consultation room, once weekly, 30–40 min per session, scheduled on the same day as the outpatient visit for convenience.
Family group psychotherapy: implemented in the mental health activity room, family-based, once every 2 weeks, 90 min per session.
Family psychological knowledge workshops: held in the mental health education room, once every 2 weeks, 90 min per session. Flexible scheduling was allowed based on family availability.

**Figure 1:**
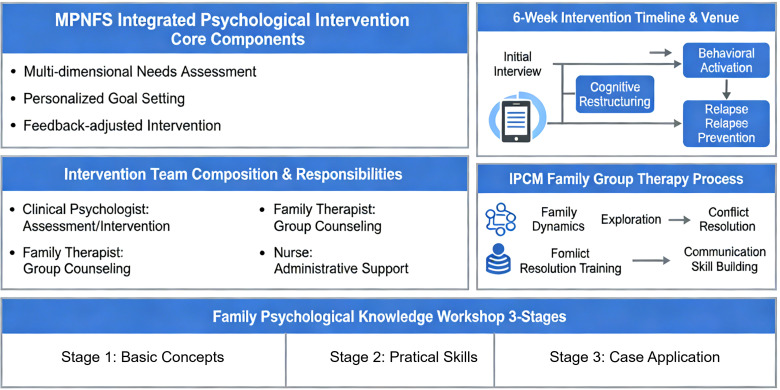
Schematic overview of the MPNFS-based integrative intervention. The figure summarizes the structure, core components, delivery format, and timeline of the MPNFS-based integrative intervention, including individual psychological counseling, family group psychotherapy, and family psychological knowledge workshops.

All sessions followed a structured protocol with predefined objectives, standardized session content, and specific exercises to ensure replicability. Intervention delivery was documented in session logs, and any deviations from the protocol were recorded and reviewed by the head nurse to maintain fidelity. The overall structure, components, delivery format, and timeline of the MPNFS-based integrative intervention are summarized in **Figure 1**.

#### Individual psychological counseling

Cognitive Behavioral Therapy (CBT), evidence-based for anxiety disorders, was adopted with the following standardized protocol:

Establishment of therapeutic alliance: Explanation of confidentiality principles and exceptions to build trust.
Psychoeducation: Illustration of the cognitive-behavioral model of anxiety to enhance treatment motivation.
Negative automatic thought management: Identification, evaluation, and development of coping strategies for dysfunctional automatic thoughts.
Belief restructuring: Recognition, challenging, and modification of negative intermediate beliefs and core schemas.
Session integration: Summary of key content, reflection on emerging issues, and goal-setting for post-intervention adaptation.

All CBT sessions were delivered by trained psychiatric nurses under supervision, and adherence to the protocol was periodically reviewed.

#### Family group psychotherapy

Integrative Problem-Centered Metaframeworks (IPCM) therapy was used to enhance family intimacy, following this structured process:

Relationship building: Establishing rapport with the adolescent and family members.
Problem exploration: Collaborative identification of specific life events triggering family conflict or adolescent anxiety.
Emotional expression: Encouraging open communication of perspectives and safe disclosure of private distress.
Misunderstanding resolution: Clarifying perceptual biases and exploring positive responses among family members.
Termination criterion: Discontinuation when family conflict is reduced and family functioning is improved.

All sessions were led by a full-time psychologist, with attendance and session content documented to ensure protocol adherence.

#### Family psychological knowledge workshops

Workshops were designed based on the three-level model of parent-child communication to improve communication competence and perceived social support. Each workshop followed a three-phase structure: ①Theoretical instruction on the workshop theme; ②Practical exercises using techniques such as empty-chair technique, role-playing, and scenario simulation; ③Feedback and guidance: Participants (adolescents and family members) shared experiences and challenges, followed by targeted comments and improvement suggestions from the psychologist. Specific workshop themes are detailed in **Table 1**.

**Table 1:** Themes of family psychological knowledge workshops.

Session	Main theme	Sub-themes
1	Re-recognizing parent-child communication	Definition of parent-child communication; Core components of effective communication; Impact of communication quality on adolescent mental health
2	Parent-child communication competence and quality	Assessment of communication competence; Strategies to enhance active listening and emotional validation; Resolution of communication barriers
3	Parent-child communication system	Structure of family communication systems; Interaction between communication competence, quality, and system dynamics; Building adaptive family communication norms

Workshop content, exercises, and session logs were standardized, and fidelity was monitored by the intervention team to ensure consistency across families.

### Outcome measures

Data were collected via questionnaires administered by trained psychiatric nurses before and immediately after the 6-week intervention. To ensure data quality and consistency, all nurses involved in data collection received unified training prior to study initiation. Standardized instructions were provided to explain the purpose and completion method of the questionnaires. Completed questionnaires were collected on-site, with immediate checks for missing or erroneous entries (supplemented or corrected on the spot if necessary). All data were double-entered and cross-checked prior to statistical analysis.

#### Psychological symptoms

Anxiety severity: Assessed using the SAS, a 20-item scale scored on a 4-point Likert scale (1=rarely/none, 4=almost always). Standard scores ≥50 indicate anxiety (mild: 50–59, moderate: 60–69, severe: ≥70).
Depressive symptoms: Evaluated with the Self-Rating Depression Scale (SDS), a 20-item scale scored on a 4-point Likert scale. Standard scores ≥53 indicate depression (mild: 53–62, moderate: 63–72, severe: ≥73).

#### Self-concept

Measured by the Piers-Harris Children’s Self-Concept Scale (PHCSS), which includes 6 subscales: Behavior, Intellectual and School Status, Physical Appearance and Attributes, Anxiety, Popularity, and Happiness and Satisfaction. Scores range from 0 to 80, with higher scores indicating higher self-concept. A score below the 30th percentile denotes low self-concept.

#### Family functioning

Parenting style: Assessed using the Egna Minnen av Barndoms Uppfostran (EMBU), a 53-item scale with 11 factors. Paternal parenting style includes 6 factors (emotional warmth and understanding, severe punishment, over-interference, preference, rejection and denial, over-protection); maternal parenting style includes 5 factors (emotional warmth and understanding, over-interference and protection, rejection and denial, severe punishment, preference).
Family intimacy: Evaluated with the Chinese Version of Family Adaptability and Cohesion Scale II (FACESII-CV), a 30-item scale (16 items for cohesion, 14 items for adaptability) scored on a 5-point Likert scale (1=never, 5=always). Total scores range from 30 to 150, with higher scores indicating greater family intimacy. Cronbach’s α was 0.92 in this study.

#### Social support and quality of life

Perceived social support: Measured by the Perceived Social Support Scale (PSSS), a 12-item scale with 3 dimensions (family support, friend support, other support) scored on a 7-point Likert scale (1=strongly disagree, 7=strongly agree). Total scores range from 12 to 84, with higher scores indicating stronger perceived support. Cronbach’s α was 0.85 in this study.
Quality of life: Assessed using the Short Form 36 Health Survey (SF-36), which evaluates 8 domains (physical functioning, role-physical, bodily pain, general health, vitality, social functioning, role-emotional, mental health). Higher scores indicate better quality of life.

### Statistical analysis

All analyses were conducted using SPSS 27.0 and R 4.3. Continuous variables were assessed for normality using the Shapiro-Wilk test and are presented as mean ± SD or median (IQR) as appropriate; categorical variables are expressed as frequencies and percentages. Between-group comparisons were performed using independent-samples t-tests or Mann-Whitney U tests for continuous variables and χ² tests for categorical variables. Linear mixed-effects models with random intercepts for participants were used for all primary and secondary outcomes. Missing data were addressed using multiple imputation under the assumption of missing at random (MAR), incorporating baseline scores, group allocation, and relevant covariates; analyses were conducted on all imputed datasets, with sensitivity analyses in participants who completed the full 6-week intervention (per-protocol analysis) to assess robustness. To control for inflation of Type I error due to multiple testing, false discovery rate (FDR) correction was applied and adjusted *P*-values are reported. Two-tailed P-values <0.05 were considered statistically significant.

## Results

### Missing data and sensitivity analysis

Missing data occurred in 12 participants (6.1%) during the 6-week follow-up, with rates ranging from 1.5% to 3.6% across outcome measures. Baseline comparisons between completers (n = 184) and non-completers (n = 12) revealed no significant differences in demographic characteristics or baseline outcome scores (all *P* > 0.05; **Table 2**), indicating that data were missing at random.

**Table 2:** Missing data and baseline comparison between completers and non-completers.

Variable	Completers (n=184)	Non-completers (n=12)	Missing data (%)	*P*
Sex, % male	48%	50%	–	0.840
Age, years	14.2 ± 2.1	14.0 ± 2.3	–	0.720
SAS score	56.3 ± 6.2	55.8 ± 5.9	2.6	0.650
SDS score	53.7 ± 5.8	54.1 ± 6.1	3.1	0.710
PHCSS total	42.1 ± 7.5	41.8 ± 7.2	3.6	0.790
EMBU total	125.3 ± 15.6	126.1 ± 14.9	2.0	0.820
FACESII-CV total	108.7 ± 12.4	109.2 ± 11.8	1.5	0.780
PSSS total	62.5 ± 9.3	61.9 ± 8.7	2.6	0.740
SF-36 total	378.4 ± 34.7	376.8 ± 35.1	3.1	0.810

SAS, Zung’s Self-Rating Anxiety Scale; SDS, Zung’s Self-Rating Depression Scale; PHCSS, Piers-Harris Children’s Self-Concept Scale; EMBU, Egna Minnen av Barndoms Uppfostran (parenting style questionnaire); FACESII-CV, Chinese Version of Family Adaptability and Cohesion Scale II; PSSS, Perceived Social Support Scale; SF-36, Short Form-36 Health Survey.

### Comparison of general data between the two groups

A total of 196 participants were enrolled, with 89 in the control group and 107 in the observation group. There were no statistically significant differences in general data between the two groups, including age, gender, duration of illness, educational level, residence, type of anxiety disorder, family structure, parental educational level, and monthly family income (all *P*>0.05), indicating comparability between the groups. Detailed data are shown in **Table 3**.

**Table 3:** Comparison of general data between the two groups.

Variables	Control group (n=89)	Observation group (n=107)	Statistical value	*P*
Age (years)	14.62 ± 2.15	14.83 ± 2.07	t=-0.683	0.495
Gender, n (%)			χ²=0.327	0.567
Male	38 (42.70)	48 (44.86)		
Female	51 (57.30)	59 (55.14)		
Duration of illness (months, M (Q1, Q3))	5.30 (3.10, 7.80)	5.50 (3.20, 8.10)	Z=-0.412	0.680
Educational level, n (%)			χ²=1.245	0.742
Primary school	12 (13.48)	15 (14.02)		
Junior high school	58 (65.17)	71 (66.36)		
Senior high school	19 (21.35)	21 (19.63)		
Residence, n (%)			χ²=0.578	0.447
Urban	53 (59.55)	61 (57.01)		
Rural	36 (40.45)	46 (42.99)		
Type of anxiety disorder, n (%)			χ²=2.136	0.343
Generalized anxiety disorder	42 (47.19)	49 (45.79)		
Social anxiety disorder	31 (34.83)	40 (37.38)		
Separation anxiety disorder	16 (17.98)	18 (16.82)		
Family structure, n (%)			χ²=1.892	0.388
Nuclear family	65 (73.03)	79 (73.83)		
Single-parent family	14 (15.73)	16 (14.95)		
Extended family	10 (11.24)	12 (11.21)		
Paternal educational level, n (%)			χ²=0.987	0.804
Junior high school and below	28 (31.46)	33 (30.84)		
Senior high school	41 (46.07)	50 (46.73)		
College and above	20 (22.47)	24 (22.43)		
Maternal educational level, n (%)			χ²=1.563	0.458
Junior high school and below	30 (33.71)	37 (34.58)		
Senior high school	40 (44.94)	49 (45.79)		
College and above	19 (21.35)	21 (19.63)		
Monthly family income (yuan, n (%))			χ²=2.318	0.314
<3000	22 (24.72)	25 (23.36)		
3000-5000	45 (50.56)	57 (53.27)		
>5000	22 (24.72)	25 (23.36)		

### Comparison of SAS and SDS scores between the two groups

**Figure 2:**
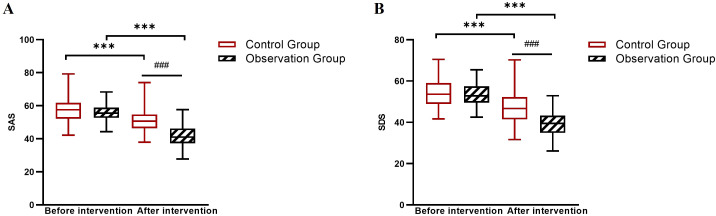
Box plots of SAS and SDS scores before and after intervention in the control and observation groups. **(A)** SAS scores, **(B)** SDS scores. ***P < 0.001 between groups (observation group *vs*. control group at post-intervention); ###*P* < 0.001 for within-group comparison (pre- *vs*. post-intervention in the observation group).

At baseline, anxiety and depression scores were comparable between groups. After 6 weeks, both groups showed reductions, with the observation group demonstrating larger within-group improvements (SAS: Δ = -14.4; SDS: Δ = -14.2) compared to the control group (SAS: Δ = -6.9; SDS: Δ = -6.8). Linear mixed-effects model analysis confirmed significant Group × Time interactions for both measures (both *P* < 0.001; **Table 4**; **Figure 2**).

**Table 4:** Linear mixed-effects model analysis of SAS and SDS scores.

Variable	SAS		SDS	
	Standardized β (SE)	*P* (FDR)	Standardized β (SE)	*P* (FDR)
Intercept	5.62 (0.05)	<0.001	5.68 (0.05)	<0.001
Time (Post *vs* Pre)	-0.60 (0.05)	<0.001	-0.59 (0.05)	<0.001
Group (Observation *vs* Control)	0.03 (0.03)	0.325	0.03 (0.03)	0.329
Group × Time	-0.65 (0.05)	<0.001	-0.64 (0.05)	<0.001

SAS, Zung Self-Rating Anxiety Scale; SDS, Zung Self-Rating Depression Scale. Values are standardized regression coefficients (β) with standard errors (SE) derived from linear mixed-effects models including random intercepts for participants. Time × Group represents the intervention effect. *P*-values were adjusted for multiple comparisons using the Benjamini–Hochberg false discovery rate (FDR) procedure. *P* < 0.05 indicates statistical significance.

### Comparison of PHCSS scores between the two groups

**Figure 3:**
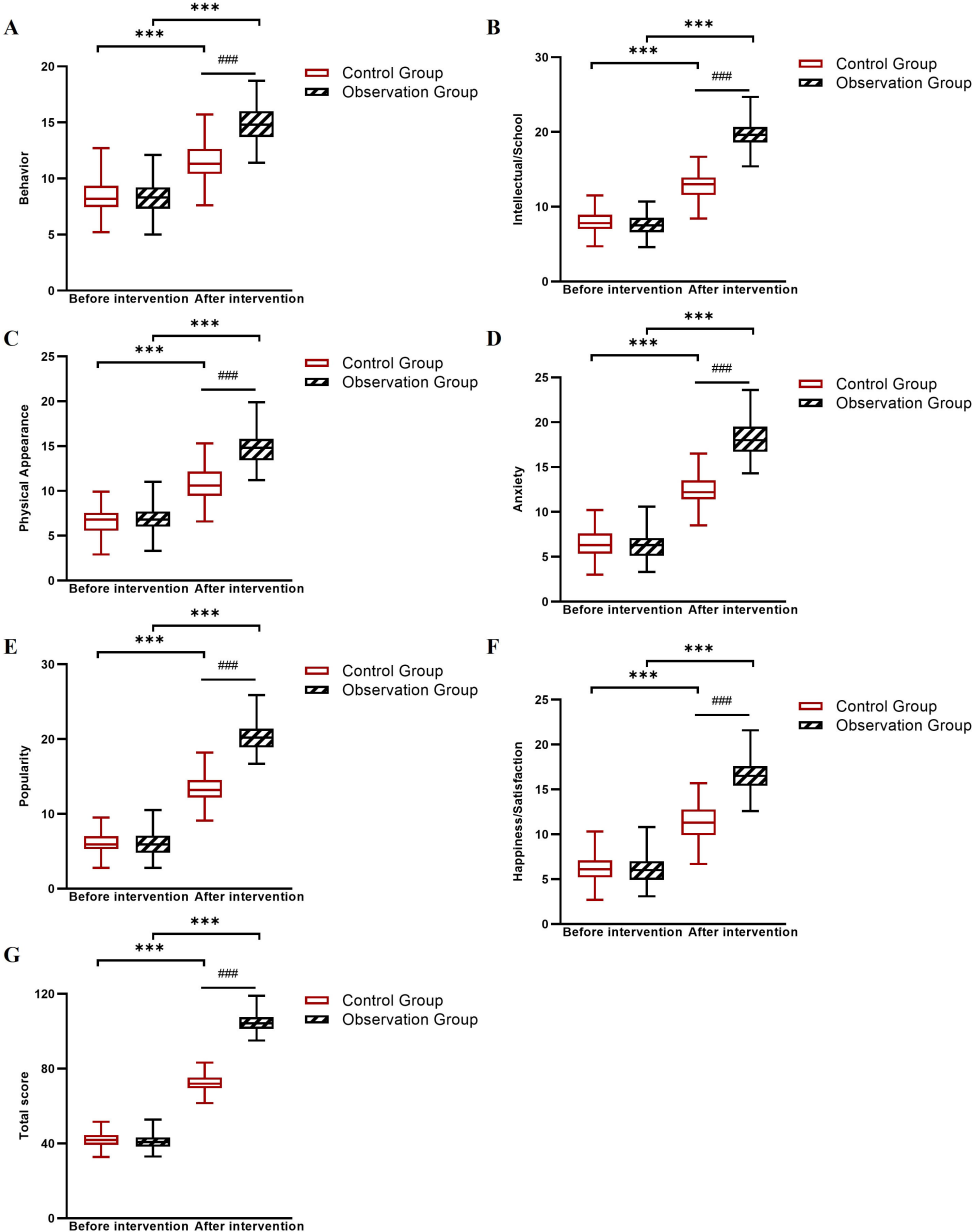
Changes in PHCSS subscale scores before and after intervention in the control and observation groups. **(A)** Behavior scores, **(B)** Intellectual and school status scores, **(C)** Physical appearance and attributes scores, **(D)** Anxiety scores, **(E)** Popularity scores, **(F)** Happiness and satisfaction scores, and **(G)** Total scores. ***P < 0.001 between groups (observation group *vs*. control group at post-intervention); ###*P* < 0.001 for within-group comparison (pre- *vs*. post-intervention in the observation group).

Baseline self-concept scores were comparable between groups. After 6 weeks, all PHCSS subscale and total scores increased in both groups, with the observation group showing substantially larger improvements (total score Δ = +34.3) than the control group (total score Δ = +16.3). Linear mixed-effects model analysis revealed significant Group × Time interactions for all subscales and the total score (all *P* < 0.001; **Table 5**; **Figure 3**).

**Table 5:** Linear mixed-effects model analysis of PHCSS subscale and total scores.

Variable	Behavior		Intellectual/School		Physical appearance		Anxiety		Popularity		Happiness/Satisfaction		Total score	
	Standardized β (SE)	*P* (FDR)	Standardized β (SE)	*P* (FDR)	Standardized β (SE)	*P* (FDR)	Standardized β (SE)	*P* (FDR)	Standardized β (SE)	*P* (FDR)	Standardized β (SE)	*P* (FDR)	Standardized β (SE)	*P* (FDR)
Intercept	0.92 (0.02)	<0.001	0.88 (0.02)	<0.001	0.76 (0.02)	<0.001	0.71 (0.02)	<0.001	0.69 (0.02)	<0.001	0.68 (0.02)	<0.001	4.62 (0.05)	<0.001
Time (Post *vs* Pre)	0.35 (0.03)	<0.001	0.57 (0.04)	<0.001	0.44 (0.03)	<0.001	0.69 (0.04)	<0.001	0.79 (0.04)	<0.001	0.56 (0.03)	<0.001	1.84 (0.06)	<0.001
Group (Observation *vs* Control)	0.03 (0.02)	0.230	0.05 (0.02)	0.180	0.02 (0.02)	0.320	0.03 (0.02)	0.220	0.05 (0.02)	0.065	0.03 (0.02)	0.220	0.03 (0.02)	0.180
Group × Time	0.44 (0.03)	<0.001	0.74 (0.03)	<0.001	0.47 (0.03)	<0.001	0.66 (0.04)	<0.001	0.80 (0.03)	<0.001	0.62 (0.03)	<0.001	2.03 (0.06)	<0.001

PHCSS, Piers-Harris Children’s Self-Concept Scale. Values are standardized regression coefficients (β) with standard errors (SE) derived from linear mixed-effects models including random intercepts for participants, adjusted for baseline PHCSS subscale or total scores. Group × Time represents the intervention effect. *P*-values were adjusted for multiple comparisons across PHCSS subscales and total score using the Benjamini–Hochberg FDR method. *P* < 0.05 indicates statistical significance.

### Comparison of EMBU scores between the two groups

At baseline, parenting style factors were comparable between groups. After 6 weeks, the observation group demonstrated greater improvements across multiple parenting domains. For paternal parenting, larger improvements were observed in emotional warmth (Δ = +21.9 *vs*. +11.8), punishment severity (Δ = -7.4 *vs*. -3.7), and rejection/denial (Δ = -7.2 *vs*. -3.6). For maternal parenting, greater improvements were observed in over-interference/protection (Δ = -19.4 *vs*. -9.7) and emotional warmth (Δ = +1.5 *vs*. +0.7). Linear mixed-effects model analysis confirmed significant Group × Time interactions for these factors (all P < 0.05; **Tables 6** and **7**), while no significant interactions were found for other EMBU factors (all *P* > 0.05).

**Table 6:** Linear mixed-effects model analysis of paternal EMBU scores.

Variable	Emotional warmth		Punishment severity		Rejection/denial		Over-interference		Over-protection		Preference	
	Standardized β (SE)	*P* (FDR)	Standardized β (SE)	*P* (FDR)	Standardized β (SE)	*P* (FDR)	Standardized β (SE)	*P* (FDR)	Standardized β (SE)	*P* (FDR)	Standardized β (SE)	*P* (FDR)
Intercept	5.34 (0.05)	<0.001	1.78 (0.03)	<0.001	1.49 (0.02)	<0.001	3.20 (0.04)	<0.001	4.09 (0.04)	<0.001	1.08 (0.02)	<0.001
Time (Post *vs* Pre)	1.33 (0.06)	<0.001	-0.42 (0.04)	<0.001	-0.41 (0.04)	<0.001	0.02 (0.04)	0.580	0.01 (0.04)	0.800	0.00 (0.02)	0.950
Group (Observation *vs* Control)	1.14 (0.06)	<0.001	-0.43 (0.04)	<0.001	-0.41 (0.03)	<0.001	0.01 (0.04)	0.750	0.01 (0.04)	0.780	0.00 (0.02)	0.960
Group × Time	1.14 (0.06)	<0.001	-0.42 (0.04)	<0.001	-0.41 (0.03)	<0.001	0.02 (0.03)	0.600	0.01 (0.04)	0.750	0.00 (0.02)	0.950

EMBU, Egna Minnen av Barndoms Uppfostran (paternal parenting style). Values are standardized regression coefficients (β) with standard errors (SE) derived from linear mixed-effects models including random intercepts for participants and adjusted for baseline EMBU subscale scores. Group × Time represents the intervention effect. *P*-values were adjusted for multiple comparisons using the FDR method. *P* < 0.05 indicates statistical significance.

**Table 7:** Linear mixed-effects model analysis of maternal EMBU scores.

Variable	Emotional warmth		Over-interference/Protection		Rejection/denial		Punishment severity		Preference	
	Standardized β (SE)	*P* (FDR)	Standardized β (SE)	*P* (FDR)	Standardized β (SE)	*P* (FDR)	Standardized β (SE)	*P* (FDR)	Standardized β (SE)	*P* (FDR)
Intercept	5.44 (0.05)	<0.001	4.02 (0.04)	<0.001	1.36 (0.02)	<0.001	1.46 (0.02)	<0.001	1.05 (0.02)	<0.001
Time (Post *vs* Pre)	0.08 (0.03)	0.023	-1.09 (0.05)	<0.001	0.01 (0.02)	0.650	0.01 (0.02)	0.700	0.01 (0.02)	0.580
Group (Observation *vs* Control)	0.09 (0.03)	0.015	-1.10 (0.04)	<0.001	0.01 (0.02)	0.600	0.01 (0.02)	0.650	0.01 (0.02)	0.540
Group × Time	0.09 (0.03)	0.012	-1.09 (0.04)	<0.001	0.01 (0.02)	0.590	0.01 (0.02)	0.640	0.01 (0.02)	0.530

EMBU, Egna Minnen av Barndoms Uppfostran (maternal parenting style). Values are standardized regression coefficients (β) with standard errors (SE) derived from linear mixed-effects models including random intercepts for participants and adjusted for baseline EMBU subscale scores. Group × Time represents the intervention effect. *P*-values were adjusted for multiple comparisons using the FDR method. *P* < 0.05 indicates statistical significance.

### Comparison of family intimacy, social support scores, and SF-36 scores between the two groups

**Figure 4:**
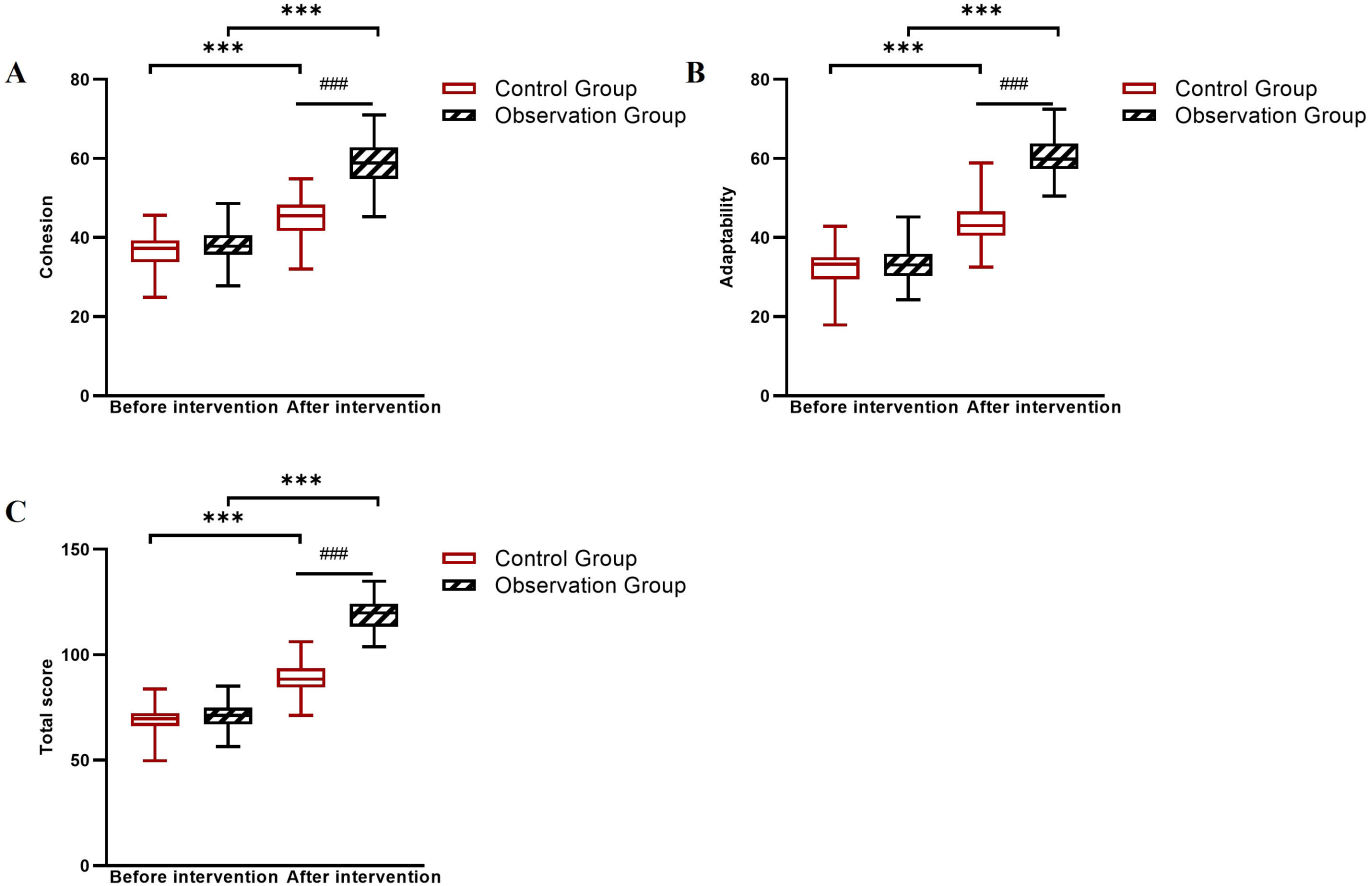
Changes in FACESII-CV subscale scores before and after intervention in the control and observation groups. **(A)** Cohesion dimension scores, **(B)** Adaptability dimension scores, and **(C)** Total scores. ***P < 0.001 between groups (observation group *vs*. control group at post-intervention); ###*P* < 0.001 for within-group comparison (pre- *vs*. post-intervention in the observation group).

**Figure 5:**
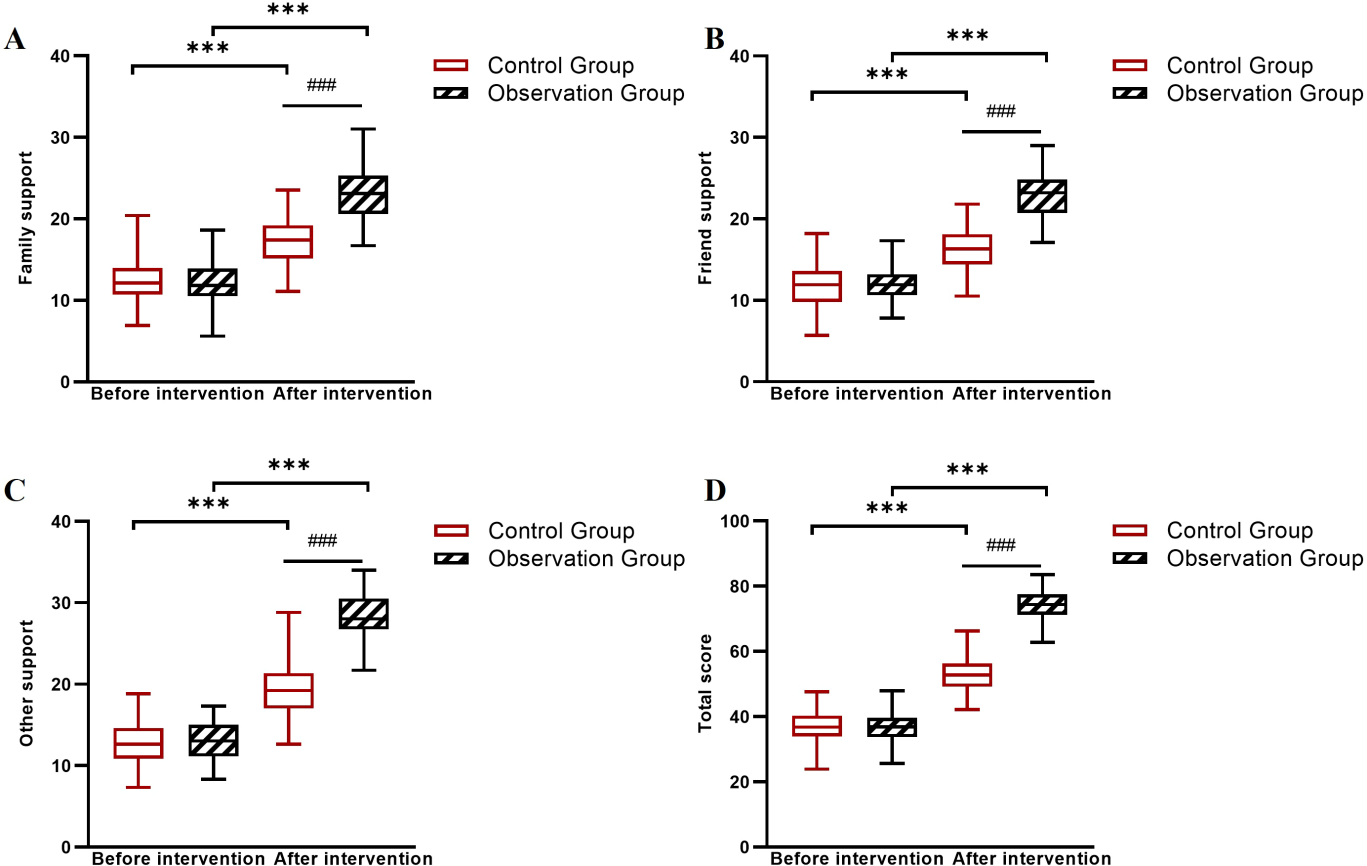
Changes in PSSS subscale scores before and after intervention in the control and observation groups. **(A)** Family support scores, **(B)** Friend support scores, **(C)** Other support scores, and **(D)** Total scores. ***P < 0.001 between groups (observation group *vs*. control group at post-intervention); ###*P* < 0.001 for within-group comparison (pre- *vs*. post-intervention in the observation group).

**Figure 6:**
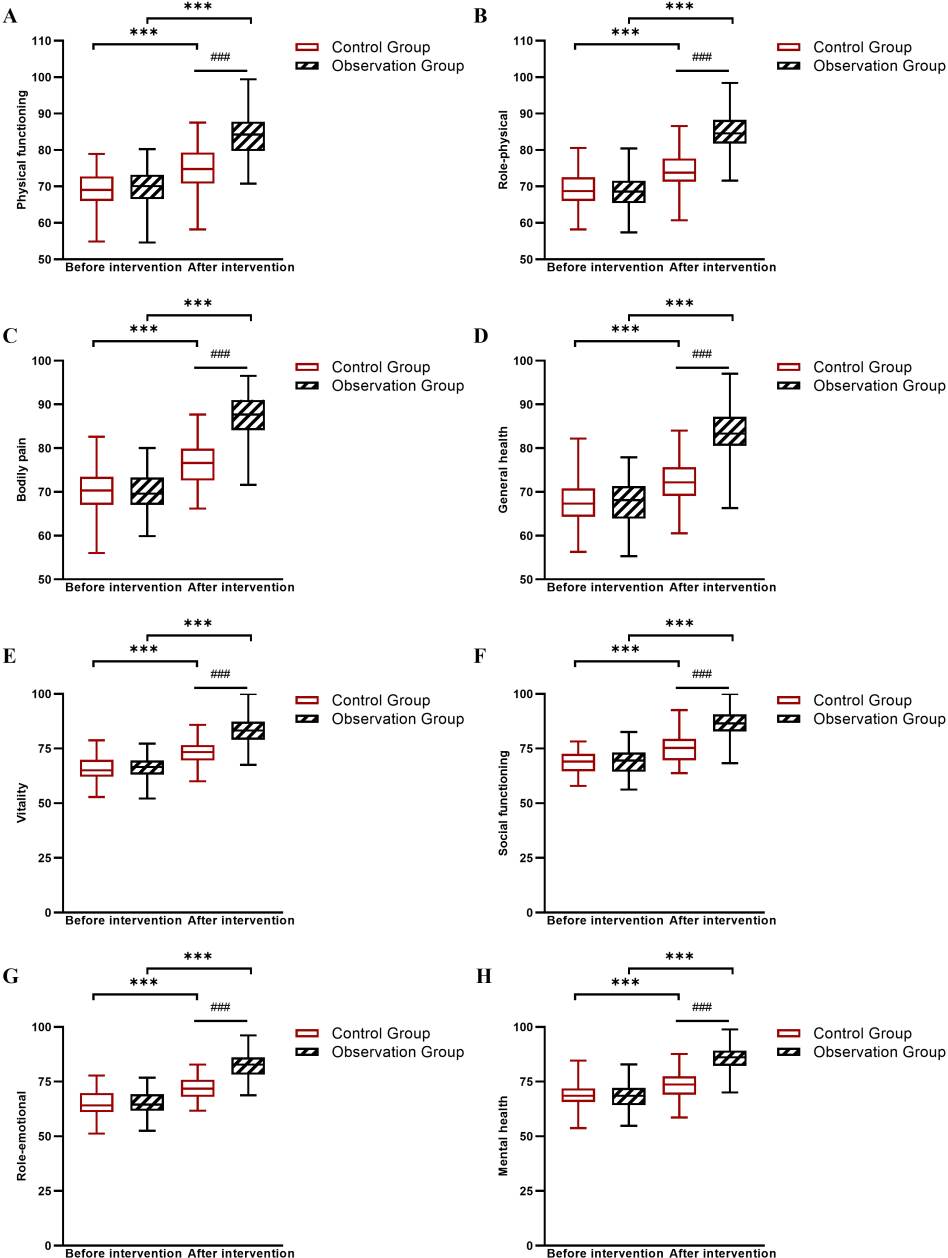
Changes in SF-36 subscale scores before and after intervention in the control and observation groups. **(A)** Physical functioning scores, **(B)** Role-physical scores, **(C)** Bodily pain scores, **(D)** General health scores, **(E)** Vitality scores, **(F)** Social functioning scores, **(G)** Role-emotional scores, and **(H)** Mental health scores. ***P < 0.001 between groups (observation group *vs*. control group at post-intervention); ###*P* < 0.001 for within-group comparison (pre- *vs*. post-intervention in the observation group).

Baseline scores for family intimacy, social support, and quality of life were comparable between groups. After 6 weeks, the observation group showed markedly larger improvements across all these measures: family intimacy (FACESII-CV total Δ = +65.0 *vs*. +17.6), perceived social support (PSSS total Δ = +31.8 *vs*. +11.6), and all SF-36 quality of life domains (observation group Δ range: +15.5 to +17.9; control group Δ range: +5.6 to +6.6). Linear mixed-effects model analysis confirmed significant Group × Time interactions for all measures (all *P* < 0.001; **Tables 8**–**10**, **Figures 4**–**6**).

**Table 8:** Linear mixed-effects model analysis of FACESII-CV scores.

Variable	Cohesion		Adaptability		Total score	
	Standardized β (SE)	*P* (FDR)	Standardized β (SE)	*P* (FDR)	Standardized β (SE)	*P* (FDR)
Intercept	4.30 (0.05)	<0.001	3.71 (0.04)	<0.001	8.01 (0.06)	<0.001
Time (Post *vs* Pre)	0.81 (0.06)	<0.001	1.18 (0.05)	<0.001	1.98 (0.08)	<0.001
Group (Observation *vs* Control)	0.03 (0.04)	0.420	0.03 (0.04)	0.380	0.06 (0.06)	0.320
Group × Time	1.50 (0.05)	<0.001	1.87 (0.05)	<0.001	5.34 (0.07)	<0.001

FACESII-CV, Family Adaptability and Cohesion Evaluation Scale, Chinese Version. Values are standardized regression coefficients (β) with standard errors (SE) derived from linear mixed-effects models including random intercepts for participants and adjusted for baseline FACESII-CV subscale or total scores. Group × Time represents the intervention effect. *P*-values were adjusted for multiple comparisons using the FDR method. *P* < 0.05 indicates statistical significance.

**Table 9:** Linear mixed-effects model analysis of social support (PSSS) scores.

Variable	Family support		Friend support		Other support		Total score	
	Standardized β (SE)	*P* (FDR)	Standardized β (SE)	*P* (FDR)	Standardized β (SE)	*P* (FDR)	Standardized β (SE)	*P* (FDR)
Intercept	1.40 (0.03)	<0.001	1.33 (0.02)	<0.001	1.44 (0.02)	<0.001	4.17 (0.05)	<0.001
Time (Post *vs* Pre)	0.51 (0.04)	<0.001	0.52 (0.03)	<0.001	0.71 (0.04)	<0.001	1.31 (0.06)	<0.001
Group (Observation *vs* Control)	0.03 (0.03)	0.320	0.03 (0.03)	0.330	0.05 (0.03)	0.270	0.06 (0.05)	0.220
Group × Time	0.62 (0.03)	<0.001	0.68 (0.03)	<0.001	1.03 (0.04)	<0.001	2.28 (0.07)	<0.001

PSSS, Perceived Social Support Scale. Values are standardized regression coefficients (β) with standard errors (SE) derived from linear mixed-effects models including random intercepts for participants and adjusted for baseline PSSS subscale or total scores. Group × Time represents the intervention effect. *P*-values were adjusted for multiple comparisons using the FDR method. *P* < 0.05 indicates statistical significance.

**Table 10:** Linear mixed-effects model analysis of quality of life (SF-36) scores.

Variable	Physical functioning		Role-physical		Bodily pain		General health		Vitality		Social functioning		Role-emotional		Mental health	
	Standardized β (SE)	*P* (FDR)	Standardized β (SE)	*P* (FDR)	Standardized β (SE)	*P* (FDR)	Standardized β (SE)	*P* (FDR)	Standardized β (SE)	*P* (FDR)	Standardized β (SE)	*P* (FDR)	Standardized β (SE)	*P* (FDR)	Standardized β (SE)	*P* (FDR)
Intercept	7.86 (0.05)	<0.001	7.74 (0.05)	<0.001	7.90 (0.05)	<0.001	7.58 (0.04)	<0.001	7.49 (0.05)	<0.001	7.72 (0.05)	<0.001	7.36 (0.05)	<0.001	7.65 (0.05)	<0.001
Time (Post *vs* Pre)	0.65 (0.04)	<0.001	0.63 (0.04)	<0.001	0.72 (0.04)	<0.001	0.65 (0.04)	<0.001	0.73 (0.04)	<0.001	0.74 (0.04)	<0.001	0.72 (0.04)	<0.001	0.71 (0.04)	<0.001
Group (Observation *vs* Control)	0.05 (0.03)	0.240	0.06 (0.03)	0.200	0.03 (0.04)	0.280	0.05 (0.03)	0.230	0.06 (0.04)	0.200	0.05 (0.03)	0.240	0.03 (0.04)	0.290	0.06 (0.03)	0.210
Group × Time	1.09 (0.05)	<0.001	1.14 (0.05)	<0.001	1.19 (0.05)	<0.001	1.18 (0.05)	<0.001	1.24 (0.05)	<0.001	1.27 (0.05)	<0.001	1.23 (0.05)	<0.001	1.21 (0.05)	<0.001

SF-36, Short Form-36 Health Survey. Values are standardized regression coefficients (β) with standard errors (SE) derived from linear mixed-effects models including random intercepts for participants and adjusted for baseline SF-36 subscale scores. Group × Time represents the intervention effect. *P*-values were adjusted for multiple comparisons using the FDR method. *P* < 0.05 indicates statistical significance.

## Discussion

This study systematically evaluates the efficacy of the MPNFS-based integrative psychological intervention in adolescents with anxiety disorders, comparing it with routine outpatient management. The findings demonstrate that the MPNFS model produces superior outcomes across multiple domains—including reduction of anxiety and depressive symptoms, enhancement of self-concept, optimization of family functioning, strengthening of perceived social support, and improvement in quality of life—highlighting the value of a structured, multi-component approach in addressing the complex needs of this population.

A key strength of the MPNFS model lies in its integration of evidence-based modalities, which overcome the limitations of fragmented, single-focus interventions (14). The observation group exhibited significant reductions in anxiety (SAS score: *P* < 0.001) and depressive symptoms (SDS score: *P* < 0.001) compared to the control group. These findings reflect the synergistic effects of combining CBT and pharmacotherapy, which target both psychological and physiological mechanisms of anxiety. Specifically, CBT may modulate maladaptive cognitive appraisals and enhance emotion regulation, while pharmacotherapy may normalize neurochemical imbalances, together producing greater symptom reduction than either modality alone (15, 16).

Notably, the MPNFS model’s emphasis on family-centered care addresses a critical factor in adolescent anxiety identified in existing literature: familial interaction patterns. The observation group demonstrated significant improvements in family functioning, including increased paternal emotional warmth, reduced paternal punishment and rejection, and decreased maternal over-interference and protection (all *P* < 0.001), alongside elevated family intimacy (*P* < 0.001). These changes are attributed to IPCM therapy and structured parent-child communication workshops, which foster open communication, conflict resolution, and practical skills in emotional validation (17). Such family-focused interventions likely reduce stress reactivity in adolescents by improving relational security (18), consistent with prior evidence linking parental rearing styles to anxiety outcomes (19).

The intervention also enhanced self-concept and perceived social support. The observation group showed significantly higher PHCSS scores across all subscales (all *P* < 0.001), indicating improved self-perception and interpersonal confidence. These improvements suggest that the MPNFS model may strengthen adolescents’ self-efficacy and coping strategies, enabling them to better manage anxiety-provoking situations. Concurrently, perceived social support increased significantly (*P* < 0.001), reflecting the effectiveness of family workshops and social resource mobilization. By actively involving family members and promoting engagement with supportive peers and community resources, the intervention appears to create a more robust psychosocial network, which can buffer against stress and reduce the likelihood of symptom relapse. These findings are consistent with studies showing that integrative interventions enhance self-concept and social connectedness, which act as protective factors against persistent anxiety in adolescents (20, 21). Moreover, improvements in perceived social support may facilitate more adaptive social interactions, further reinforcing self-esteem and emotional resilience.

Quality of life improvements were also observed, with higher scores in all SF-36 domains (all *P* < 0.001). This demonstrates that the MPNFS model not only alleviates psychological symptoms but also produces measurable functional benefits in physical, emotional, and social domains, highlighting the holistic impact of the intervention. Such multidimensional improvements underscore the clinical relevance of structured, multi-component interventions in outpatient settings, particularly in addressing the complex interplay between symptom reduction, psychosocial functioning, and overall well-being (14). These findings support the potential for broader application of the MPNFS model in real-world clinical practice, emphasizing the importance of integrating psychological, familial, and social support components to achieve sustainable improvements in adolescent mental health.

Several limitations should be acknowledged explicitly. First, the retrospective design may introduce selection bias, as group assignment was not randomized and could reflect clinical or familial preferences. Second, the absence of long-term follow-up limits conclusions regarding the sustainability of intervention effects. Third, reliance on self-report instruments may introduce response bias. Fourth, this study did not examine differential effects across anxiety subtypes (e.g., generalized *vs*. social anxiety), which may influence responsiveness to intervention. Finally, some structural and linguistic inconsistencies remain, potentially affecting clarity. These limitations may impact the generalizability of our findings to other populations or clinical settings, and caution should be exercised when extrapolating results beyond the study sample.

Despite these limitations, this study provides novel evidence on the application of the MPNFS model in adolescents, demonstrating a structured, replicable approach that simultaneously targets symptomatic, familial, and social dimensions of anxiety. By linking observed outcomes to prior research, this work reinforces the theoretical and practical rationale for multi-component interventions and underscores the importance of integrating pharmacotherapy, CBT, family-centered care, and social support mobilization in adolescent mental health care. Future research should include prospective, randomized designs with larger samples, long-term follow-up, objective outcome measures, and subgroup analyses by anxiety subtype. Such studies could further clarify the mechanisms of the MPNFS model and optimize its implementation in diverse clinical contexts.

This study demonstrates that the MPNFS-based integrative intervention significantly improves anxiety and depressive symptoms, self-concept, family functioning, social support, and quality of life in adolescents with anxiety disorders. These findings support the clinical potential of this multi-component approach in addressing both psychological and psychosocial dimensions of adolescent anxiety. While the results are promising, longitudinal research and randomized controlled trials are needed to confirm the sustainability of these effects and further validate the MPNFS model.

## Data Availability

The original contributions presented in the study are included in the article/supplementary material. Further inquiries can be directed to the corresponding author.
